# Postoperative Outcomes of Distal Pancreatectomy for Retroperitoneal Sarcoma Abutting the Pancreas in the Left Upper Quadrant

**DOI:** 10.3389/fonc.2021.792943

**Published:** 2021-12-20

**Authors:** Kyeong Deok Kim, Kyo Won Lee, Ji Eun Lee, Jeong Ah Hwang, Sung Jun Jo, Jinseob Kim, So Hee Lim, Jae Berm Park

**Affiliations:** ^1^ Department of Surgery, Samsung Medical Center, Sungkyunkwan University School of Medicine, Seoul, South Korea; ^2^ Department of Radiology, Soonchunhyang University College of Medicine, Bucheon Hospital, Bucheon, South Korea; ^3^ Department of Radiology and Center for Imaging Science, Samsung Medical Center, Sungkyunkwan University School of Medicine, Seoul, South Korea; ^4^ Department of Epidemiology, School of Public Health, Seoul National University, Seoul, South Korea; ^5^ Transplantation Research Center, Samsung Medical Center, Seoul, South Korea

**Keywords:** retroperitoneal sarcoma, distal pancreatectomy (DP), microscopic pancreas invasion, complication, local recurrence (LR)

## Abstract

**Background:**

En bloc resection of the tumor with adjacent organs is recommended for localized retroperitoneal sarcoma (RPS). However, resection of the pancreas is controversial because it may cause serious complications, such as pancreatic fistula or bleeding. Thus, we evaluated the outcomes of distal pancreatectomy (DP) in pancreas-abutting RPS of the left upper quadrant (LUQ).

**Methods:**

We *retrospectively* reviewed all consecutive patients who underwent surgery for RPS between September 2001 and April 2020. We selected 150 patients with all or part of their tumor located in the LUQ on preoperative computed tomography. Eighty-six patients who had tumors abutting the pancreas were finally enrolled in our study.

**Results:**

Fifty-three patients (53/86; 61.6%) were included in the non-DP group, and 33 patients (33/86; 38.4%) were included in the DP group. Total postoperative complications and complication rates for those Clavien–Dindo grade 3 or higher were similar between the non-DP group and DP group (p = 0.290 and p = 0.550). In the DP group, grade B pancreatic fistulae occurred in 18.2% (6/33) of patients, but grade C pancreatic fistulae were absent, and microscopic pancreatic invasion was noted in 42.4% (14/33) of patients. During multivariate analysis, microscopic pancreatic invasion was deemed a risk factor for local recurrence (p = 0.029). However, there were no significant differences on preoperative computed tomography findings between the pancreatic invasion and non-invasion groups.

**Conclusion:**

DP is a reasonable procedure for pancreas-abutting RPS located at the LUQ when both complications and complete resection are considered.

## Introduction

Soft tissue sarcoma is rare, accounting for less than 1% of all cancers, and retroperitoneal sarcoma (RPS) accounts for about 15% of all soft tissue sarcomas (1). RPS is usually asymptomatic and often detected after the tumor has grown large because there is no anatomical barrier around the area (2). Although surgery to address large tumors is often challenging, complete surgical resection is the only potential curative treatment for patients with localized RPS (3). The 10-year recurrence rate for extremity sarcoma is 25%, while the five-year recurrence rate for RPS is as high as 50% (4). Unlike extremity sarcoma, the tumor-related mortality of RPS is mainly due to local recurrence (LR) in the absence of distant metastasis (5). Therefore, en bloc resection with adjacent structures is recommended to increase the safety margins of RPS treatment (2). The optimal extent of resection is considered to encompass both the attainment of good oncologic outcomes and the avoidance of serious complications. Resection of the kidney and colon is accepted as a treatment for RPS due to the relatively low morbidity, but resection of the pancreas is more controversial given the accompanying high risk of morbidity and death. Li et al. reported that pancreaticoduodenectomy (PD) is a feasible way to achieve complete resection for right-sided RPS despite the many major complications that may occur (6). Bagaria et al. also reported that distal pancreatectomy (DP) for primary RPS can achieve complete resection with acceptable morbidity and oncologic outcomes (7). On the other hand, Flacs et al. reported that pancreatic resection was associated with significant postoperative morbidity and mortality, and PD should be avoided whenever possible (8).

In this study, we aimed to evaluate postoperative outcomes of DP for RPS abutting the pancreas in the left upper quadrant (LUQ) area.

## Materials and Methods

This *retrospective* study was approved by the institutional review board (IRB) of our institution (IRB no. 2021-05-125), who also waived the need for informed consent.

### Patients and Data

**Figure 1:**
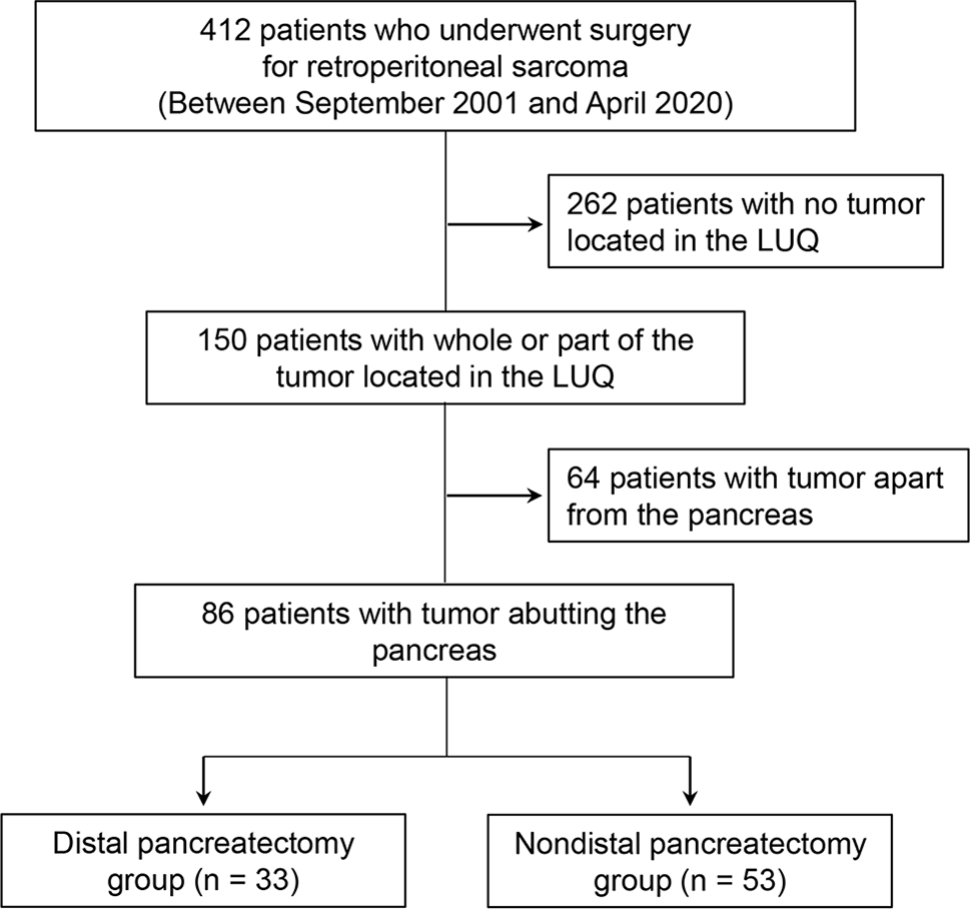
Flow diagram of patient selection criteria for our study. LUQ, left upper quadrant.

We retrospectively reviewed all consecutive patients who underwent surgery for RPS at Samsung Medical Center in Seoul, Korea, between September 2001 and April 2020, and gathered 412 patients. All patients underwent contrast-enhanced abdominal computed tomography (CT) imaging at our institution for preoperative imaging analysis and, based on the results, 150 patients with all or part of the tumor located in the LUQ area were selected. All preoperative CT images of these 150 patients were reviewed by two abdominal radiologists (J. E. L. and J. A. H., each with 10–11 years of experience in abdominal imaging) to determine whether the tumor was in contact with the pancreas. Among 150 patients, 64 with an obvious fat plane preserved between the pancreas and tumor were excluded, and 86 patients with at least some part of the tumor abutting the pancreas were enrolled in our study. The enrolled patients were then divided into two groups depending on whether they had combined DP; 53 patients (53/86; 61.6%) were included in the non-DP group, and 33 patients (33/86; 38.4%) were included in the DP group. The decision to perform DP was made at the surgeon’s discretion during surgery. **Figure 1** presents a flowchart of patient selection criteria.

As for the surgical technique, all pancreatectomy was performed including splenectomy. Also, pancreas resection was made to perform minimal pancreatic tail resection, and to achieve negative resection margin. Patients undergoing their first tumor resection were defined as cases of primary RPS, and those with repeated surgery for recurrent tumors or incomplete resection margins achieved during the first surgery were defined as otherwise. The completeness of resection was classified as macroscopically complete (R0/1) or incomplete (R2). Histopathologic findings were confirmed by a pathologist specializing in soft tissue sarcomas and classified based on the fourth edition of the World Health Organization’s classification of soft tissue tumors (9).

Tumor grade was classified according to the *Fédération Nationale des Centres de Lutte Contre le Cancer* (FNCLCC) grading system, which considers three factors: differentiation, mitotic count, and tumor necrosis. Morbidity data were collected regarding the presence and severity of any postoperative complication before discharge. Complications were classified according to the Clavien–Dindo classification scheme (10), and severe complications were considered to be those grade 3a or higher. Pancreas-related complications, such as postoperative pancreatic fistula (POPF), were classified according to the International Study Group for Pancreatic Fistula score (11), and grades B and C POPF were considered “clinically significant” (12).

### Preoperative CT Imaging Analysis of Patients in the DP Group

**Figure 2:**
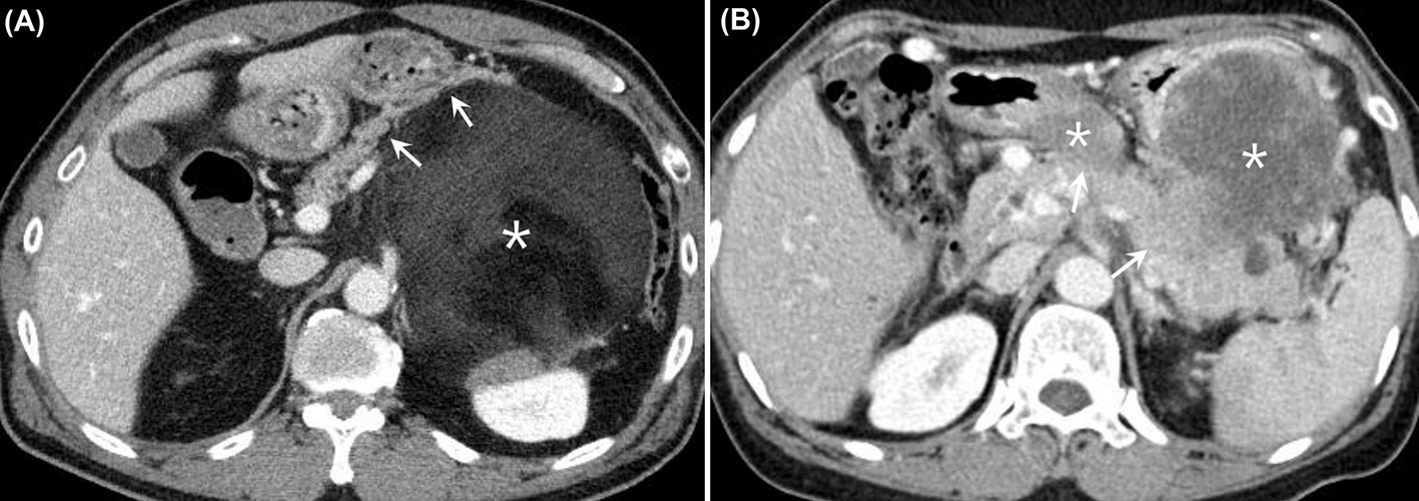
**(A)** Contrast-enhanced CT images from a 61-year-old man who had a well-differentiated liposarcoma in the LUQ of the abdomen. There was a huge heterogeneous retroperitoneal mass with predominant fat attenuation (*asterisk*). The mass was found to be anteriorly displacing the pancreatic body and tail (*arrows*). The patient underwent DP along with excision of the tumor, and there was no evidence of microscopic invasion of the pancreas. **(B)** Contrast-enhanced CT images from a 60-year-old woman who had dedifferentiated liposarcoma in the LUQ of the abdomen. There were several heterogeneously enhancing retroperitoneal masses observed between the stomach and the pancreas (*asterisks*). The enhancing solid portions of the mass appeared to be directly abutting the pancreatic body and tail (*arrows*), with the fat plane disrupted. The patient underwent DP along with excision of the tumor, and there was microscopic invasion of the pancreas by the tumor. CT, computed tomography; LUQ, left upper quadrant; DP, distal pancreatectomy.

In consensus, two abdominal radiologists (J. E. L. and J. A. H.) performed more detailed evaluations of the preoperative CT imaging findings of the 33 patients in the DP group. Both radiologists sought to determine whether there were any preoperative CT imaging findings that could predict microscopic invasion of the pancreas. Three imaging findings were evaluated: the length (cm) of the tumor abutting the pancreas, the presence of pancreas parenchymal displacement by the tumor, and the presence of an enhancing solid portion of the tumor abutting the pancreas. Both readers were aware of the diagnosis of RPS, but were blinded to the detailed pathological results (**Figures 2A, B**).

### Statistical Analyses

Continuous variables are presented as mean ± standard deviation values and were compared using the Student’s t-test and one-way analysis of variance. Categorical variables are presented as number and percentage and were compared by the chi-squared test. Overall survival (OS) was defined as the interval between the date of resection to the date of death from any cause or the date of the last follow-up for patients who were still alive. Local recurrence-free survival (LRFS) was defined as the interval between the date of resection and the date of first local recurrence or the last follow-up. OS and LRFS were estimated using the Kaplan–Meier method and compared using the log-rank test. Cox proportional-hazards model analyses were used to predict patient survival and disease recurrence. A multivariate analysis was performed using the factors from the univariate analysis that were statistically significant (p < 0.05) and clinically significant factors that were not statistically significant in the univariate analysis. All tests were two-tailed, and statistical significance was defined as p < 0.05. All statistical analyses were conducted using the R version 4.0.4 software program (R Foundation for Statistical Computing, Vienna, Austria).

## Results

### Patient and Tumor Characteristics According to Resection of the Pancreas

The comparison of patient characteristics according to resection of the pancreas is presented in **Table 1**. Patient sex, body mass index (BMI), history of diabetes mellitus and hypertension, adjuvant treatment with chemotherapy and radiotherapy, vascular resection, operation time, and estimated blood loss did not differ significantly between groups. However, patient age and the number of organs resected en bloc were significantly higher in the DP group (p = 0.035 and p < 0.001, respectively).

**Table 1:** Clinicopathologic characteristics according to resection of the pancreas.

	Non-distal pancreatectomy (n = 53)	Distal pancreatectomy (n = 33)	p-value
Patient characteristics			
Age (years)	50.7 ± 14.9	57.3 ± 12.9	0.035
Sex (n, % male)	23 (43.4)	17 (51.5)	0.609
Race (n, %)			
Asian	53 (100.0)	33 (100.0)	
BMI (kg/m^2^)	22.6 ± 3.1	21.6 ± 2.5	0.095
Patient comorbidities			
Diabetes mellitus (n, %)	1 (1.9)	0 (0)	1.000
Hypertension (n, %)	11 (20.8)	7 (21.2)	1.000
Adjuvant treatment			
Chemotherapy (n, %)	9 (17.0)	8 (24.2)	0.587
Radiotherapy (n, %)	28 (52.8)	21 (63.6)	0.447
Number of organs resected en bloc	1.0 ± 0.9	3.8 ± 0.9	< 0.001
Vascular resection (n, %)	1 (1.9)	0 (0)	1.000
Operation time (min)	354.9 ± 159.2	485.2 ± 358.1	0.056
Estimated blood loss (ml)	1284.2 ± 2069.2	1542.5 ± 2624.3	0.633
Tumor characteristics			
Primary RPS (n, %)	40 (75.5)	22 (66.7)	0.523
Tumor size (mm)	242.2 ± 137.9	218.5 ± 88.8	0.334
Surgical margin (n, %)			0.735
R0/R1	43 (82.7)	29 (87.9)	
R2	9 (17.3)	4 (12.1)	
Primary tumor histology (n, %)			0.018
WDLPS	14 (26.4)	9 (27.3)	
DDLPS	18 (34.0)	21 (63.6)	
Pleomorphic LPS	2 (3.8)	2 (6.1)	
Leiomyosarcoma	8 (15.1)	0 (0)	
MPNST	3 (5.7)	0 (0)	
solitary fibrous tumor	3 (5.7)	0 (0)	
other	5 (9.4)	1 (3.0)	
FNCLCC (n, %)			0.142
Grade 1	22 (44.0)	9 (27.3)	
Grade 2	14 (28.0)	8 (24.2)	
Grade 3	14 (28.0)	16 (48.5)	

BMI, body mass index; RPS, retroperitoneal sarcoma; WDLPS, well-differentiated liposarcoma; DDLPS, dedifferentiated liposarcoma; LPS, liposarcoma; LMS, leiomyosarcoma; MPNST, malignant peripheral nerve sheath tumor; FNCLCC, Fédération Nationale des Centres de Lutte Contre le Cancer.

The comparison of tumor characteristics according to resection of the pancreas is also shown in **Table 1**. Primary RPS, tumor size, macroscopically complete resection, and tumor grade according to FNCLCC did not differ significantly between the groups, but primary tumor histology differed significantly between groups. In the non-DP group, 34.0% of patients had dedifferentiated liposarcoma (DDLPS); 26.4%, well-differentiated liposarcoma (WDLPS); 3.8%, pleomorphic liposarcoma (LPS); 15.1%, leiomyosarcoma (LMS); 5.7%, malignant peripheral nerve sheath tumor (MPNST); 5.7%, solitary fibrous tumor (SFT); and 9.4%, others. In the DP group, 63.6% of patients had DDLPS; 27.3%, WDLPS; 6.1%, pleomorphic LPS; and 3.0%, others; there were no patients with LMS, MPNST, and SFT.

### Clinical Outcomes According to Resection of the Pancreas

Rates of complications, severe complications, postoperative bleeding and transfusion, lymphatics leakage, sepsis, hospitalization in the intensive care unit (ICU), hospitalization period, and patient mortality within 30 days did not differ between the groups. POPF occurred in six patients (6/33; 18.2%) in the DP group, but all cases were grade B. In addition, postoperative bleeding in the DP group was not associated with POPF. Fourteen patients (14/33; 42.4%) in the DP group experienced microscopic pancreatic invasion (**Table 2**).

**Table 2:** Clinical outcomes according to resection of the pancreas.

	Non-distal pancreatectomy (n = 53)	Distal pancreatectomy (n = 33)	p-value
Complications (n, %)	23 (43.4)	19 (57.6)	0.290
Severe complication (n, %)	7 (13.2)	6 (18.2)	0.550
Postoperative bleeding (n, %)	8 (15.1)	5 (15.2)	1.000
Postoperative transfusion (n, %)	9 (17.0)	7 (21.2)	0.837
Lymphatics leakage (n, %)	2 (3.8)	3 (9.1)	0.367
Sepsis (n, %)	1 (1.9)	2 (6.1)	0.556
POPF			
None/biochemical leak (n, %)	NA	27 (81.8)	
Grade B (n, %)	NA	6 (18.2)	
Grade C (n, %)	NA	0 (0)	
Microscopic pancreatic invasion	NA	14 (42.4)	
Hospitalization in ICU (n, %)	3 (5.7)	0 (0)	0.282
Hospitalization period (days)	26.9 ± 23.5	30.6 ± 20.4	0.439
Patient mortality within 30 days (n, %)	1 (1.9)	2 (6.1)	0.556

POPF, postoperative pancreatic fistula; NA, not available; ICU, intensive care unit.

### Overall Survival and Recurrence-Free Survival Rates According to Resection of the Pancreas

Resection of the pancreas did not affect the OS. The one-, five-, and ten-year OS rates were 84.8%, 45.8%, and 25.0%, respectively, in the DP group and 90.5%, 59.3%, and 32.1%, respectively, in the non-DP group (p = 0.145) (**Supplementary Figure 1A**). Also, resection of the pancreas did not influence recurrence-free survival. The one-, five-, and ten-year LRFS rates were 74.8%, 37.5%, and 18.8%, respectively, in the DP group and 76.1%, 39.7%, and 18.9%, respectively, in the non-DP group (p = 0.807) (**Supplementary Figure 1B**).

### Univariate and Multivariate Analyses of Risk Factors for Overall Survival and Recurrence-Free Survival

R2 resection and FNCLCC grade 3 were significant risk factors associated with OS in both the univariate (p < 0.001 and p <.001, respectively) and multivariate (p < 0.001 and p < 0.001, respectively) analyses; however, resection of pancreas was not associated with OS in either the univariate or multivariate analysis (p = 0.172 and p= 0.232, respectively) (**Supplementary Table 1**).

Primary tumor, R2 resection, and FNCLCC grade 3 were significant risk factors associated with LR in both the univariate (p = 0.043, p = 0.014, and p = 0.047, respectively) and multivariate (p = 0.048, p = 0.017, and p = 0.044, respectively) analyses; however, resection of pancreas was not associated with LR in either the univariate or multivariate analysis (p = 0.783 and p = 0.863, respectively) (**Supplementary Table 2**).

### Overall Survival and Recurrence-Free Survival Rates According to Microscopic Pancreatic Invasion in the DP Group

**Figure 3:**
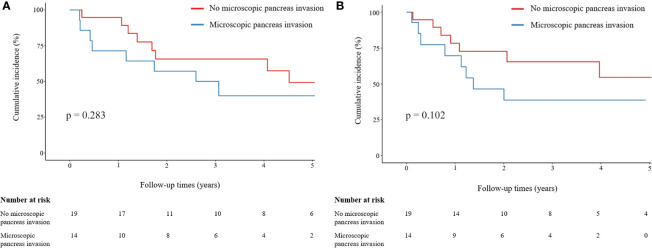
Comparison of OS and LRFS rates between patients with and without microscopic pancreatic invasion. **(A)** Comparison of OS between patients with and without microscopic pancreatic invasion. **(B)** Comparison of LRFS between patients with and without microscopic pancreatic invasion. Group comparisons were performed using Kaplan–Meier and log-rank tests. OS, overall survival; LRFS, local recurrence-free survival.

Microscopic pancreatic invasion did not affect the OS. The one-, three-, and five-year OS rates were 94.7%, 65.7%, and 49.3%, respectively, in the group without microscopic pancreatic invasion and 71.4%, 50.0%, and 40.0%, respectively, in the microscopic pancreatic invasion group (p = 0.283) (**Figure 3A**). Also, microscopic pancreatic invasion did not appear to influence recurrence-free survival in univariate analysis. The one-, three-, and five-year recurrence-free survival rates were 78.3%, 65.4%, and 54.4%, respectively, in the group without microscopic pancreatic invasion and 69.6%, 38.7%, and 38.7%, respectively, in the microscopic pancreatic invasion group (p = 0.102) (**Figure 3B**).

### Univariate and Multivariate Analyses of Risk Factors Associated With Local Recurrence and Overall Survival in Patients Who Underwent DP

In the univariate analysis, R2 resection (p = 0.014) and FNCLCC grade 3 (p = 0.012) were significant risk factors associated with LR. However, in the multivariate analysis, microscopic pancreatic invasion (p = 0.029) and FNCLCC grade 3 (p = 0.014) were significant risk factors associated with LR, while R2 resection was not (p = 0.073) (**Table 3**).

**Table 3:** Univariate and multivariate analyses of risk factors associated with local recurrence in patients who underwent DP.

Variables	Univariate		Multivariate	
	HR (95% CI)	p-value	HR (95% CI)	p-value
Age	1.02 (0.98–1.06)	0.441		
Male sex	1.02 (0.39–2.66)	0.962		
BMI	0.89 (0.73–1.10)	0.279		
Primary RPS	0.80 (0.30–2.12)	0.657		
Tumor size	1.00 (1.00–1.10)	0.598		
Microscopic pancreatic invasion	2.25 (0.83–6.09)	0.112	3.23 (1.13–9.25)	0.029
R2 resection	5.33 (1.41–20.2)	0.014	3.65 (0.89–15.09)	0.073
DDLPS	1.75 (0.61–5.00)	0.299		
FNCLCC histologic grade				
Grade 1 or 2	1 (Ref.)			
Grade 3	4.12 (1.36–12.5)	0.012	4.57 (1.35–15.42)	0.014

DP, distal pancreatectomy; BMI, body mass index; RPS, retroperitoneal sarcoma; DDLPS, dedifferentiated liposarcoma; FNCLCC, Fédération Nationale des Centres de Lutte Contre le Cancer; HR, hazard ratio; CI, confidence interval.

Meanwhile, R2 resection and FNCLCC grade 3 were significant risk factors associated with OS in both the univariate (p < 0.001 and p = 0.002, respectively) and multivariate (p = 0.009 and p = 0.003, respectively) analyses; however, microscopic pancreatic invasion was not associated with OS in either the univariate or multivariate analysis (p = 0.288 and 0.119, respectively) (**Supplementary Table 3**).

### CT Findings According to Microscopic Pancreatic Invasion

The length of the tumor abutting the pancreas, the presence of pancreas parenchymal displacement, and the presence of an enhancing solid portion of the RPS abutting the pancreas on preoperative CT imaging were not significantly associated with microscopic pancreatic invasion of the RPS in patients who underwent DP (p = 0.083, p = 1.000, and p = 0.991, respectively) (**Table 4**).

**Table 4:** CT findings according to microscopic pancreatic invasion.

	No microscopic pancreatic invasion (n = 19)	Microscopic pancreatic invasion (n = 14)	p-value
Abutment length (cm)	7.1 ± 3.2	5.2 ± 2.8	0.083
Pancreas parenchymal displacement (n, %)	18 (94.7)	13 (92.9)	1.000
Pancreas abutting the enhancing solid portion (n, %)	11 (57.9)	9 (64.3)	0.991

## Discussion

In this study, among 86 patients with tumors located in the LUQ and abutting the pancreas, 33 (33/86; 38.4%) patients underwent DP. The rates of complications and severe complications were similar between the DP and non-DP groups. POPF occurred in six patients (6/33; 18.2%) in the DP group, but all cases were grade B. Fourteen patients (14/33; 42.4%) showed microscopic pancreatic invasion in the DP group. Resection of the pancreas itself was not a risk factor associated with OS and LR. However, microscopic pancreatic invasion was a risk factor associated with LR but not OS in the DP group. Preoperative CT findings could not discriminate the presence of microscopic pancreatic invasion.

Complete resection achieving negative margins in RPS improves survival (13). However, the extent of the appropriate resection range is still being studied. Singer et al. (14) reported that contiguous organ resection without nephrectomy was a risk factor associated with death from tumor. Thus, they reported that nephrectomy may be necessary to achieve complete resection in retroperitoneal LPS. Similarly, we reported in a previous study that nephrectomy had a beneficial effect on disease-free survival in retroperitoneal LPS (15). Bonvalot et al. (16) observed that complete compartmental resection more significantly reduced the recurrence rate than simple complete resection or contiguously involved organ resection. However, in their study, the pancreas was not included in compartmental resection unless it was involved. The primary reason to consider resection of the pancreas is because the incidence of complications is high. In a systematic review in 2005, there were studies in which the morbidity rate was reported to exceed 40%, with the highest rate (of 64%) reported after DP by Knaebel et al. (17). However, Korrel et al. (18) recently found that the severe complication rate was 21.3% to 34.9% after DP, which was lower than in previous reports.

There have been few studies on the outcomes of patients undergoing DP in RPS. Keung et al. (19) retrospectively reviewed 43 cases from a single-center cohort and reported outcomes after DP in patients with non-pancreatic retroperitoneal tumors; notably, 39.5% (17/43) of their patients were indicated for RPS. In their study, the rates of complications and severe complications were 65.1% (28/43) and 23.3% (10/43), respectively. Also, POPF occurred in 32.6% (14/43) of cases, all of which were grade B (no grade C). Flacs et al. (8) retrospectively reviewed 50 cases in a dual-center study and reported outcomes of pancreatic resection for RPS. In their study, 86% (43/50) of patients underwent DP, and rates of complications and severe complications were 64% (32/50) and 28% (14/50), respectively. Also, POPF occurred in 14% (7/50) of cases, with 12% (6/50) of cases being grade B and 2% (1/50) of cases being grade C. The Trans-Atlantic Australasian Retroperitoneal Sarcoma Working Group (TARPSWG) retrospectively reviewed 280 cases in a multicenter study and reported outcomes after DP for RPS (7). In their study, rates of complications and severe complications were 62.5% (175/280) and 39.6% (111/280), respectively. Also, POPF occurred in 23.6% of cases—18.6% being grade B and 5% being grade C. In a recent meta-analysis, a total of 8,864 patients who underwent DP were included, and POPF occurred in 20.4%, with 20.2% and 1.6% being grades B and C, respectively (20). In our study, rates of complications and severe complications in the DP group were 57.6% and 18.2%, respectively, which were higher than those of 43.4% and 13.2% in the non-DP group, respectively, but not significantly so. These results suggest that the fear of greater complications in DP may not be warranted. Also, POPF occurred in 18.2% of cases, all of which were grade B. Among grade B cases, five patients required percutaneous drainage and one patient needed a pancreatic drain for more than three weeks after surgery. Also, among 27 patients without POPF, there were eight biochemical leaks. However, there were no cases of organ failure, reoperation, or death. Notably, this study achieved similar or better results relative to those of previous studies, and our results suggest that DP can be safely performed in patients with a pancreas-abutting mass located in the LUQ with respect to complications. However, performing PD in RPS is still controversial. Flacs et al. (8) reported that mortality occurred in 40% of patients who underwent PD. Thus, they suggested that PD should be avoided whenever possible. However, only five of their patients underwent PD, and the study sample size was small. On the other hand, Li et al. (6) reviewed the details of 27 patients who underwent PD for RPS and reported that rates of severe complications, POPF, and mortality were 40.7% (11/27), 29.6% (8/27), and 3.7% (1/27), respectively. Thus, they concluded that PD in RPS is a feasible way to achieve complete resection.

Studies have shown that macroscopic complete resection improves OS and local control (21, 22). Also, Bonvalot et al. (16) reported that microscopic positive margins decreased OS by 1.7-fold and increased the recurrence rate by 3.4-fold. When only patients with confirmed microscopic pancreatic invasion were analyzed among all patients with resected pancreas, Fairweather et al. (23) confirmed invasion in 42.9% (3/7), Flacs et al. (8) in 34% (17/50), and the French Sarcoma Group (24) in 15.2% (5/33), respectively. In addition, the TARPSWG analyzed only patients who underwent DP and confirmed microscopic pancreatic invasion in 38.2% (107/280) (7). Similarly, in our study, microscopic pancreatic invasion was confirmed in 42.4% (14/33) of patients who underwent DP. The TARPSWG reported that microscopic pancreatic invasion did not affect either LR or OS, but the R1 pancreatic margin was associated with LR (7). However, microscopic pancreatic invasion was deemed a risk factor associated with LR when multivariate analysis was performed in our study.

Due to widespread availability, CT has become increasingly useful in the diagnosis and preoperative evaluation of almost all anatomical diseases. However, diagnostic challenges still remain in the precise localization and detection of adjacent organ invasion in patients with RPS. Furthermore, achieving success becomes even more challenging when it comes to determining the presence or absence of microscopic organ invasion. We hypothesized that, when greater lengths of RPS are in contact with the pancreas, when the pancreas is displaced by the tumor, or when the pancreas is in contact with the enhancing solid portion of the RPS, the possibility of microscopic pancreatic invasion may increase. However, none of these findings were significant predictors for microscopic pancreatic invasion. Thus, our results show that, even using state-of-the-art CT imaging, it is difficult to preoperatively predict microscopic pancreatic invasion by RPS.

This retrospective study had some limitations. First, this was a single-center cohort of patients referred to a tertiary medical center and, due to our relatively small sample size and only a single ethnicity, the results may be misestimated. Also, the inclusion period of this study was quite long, which causes selection bias. During this period, the therapeutic approach changed and clinician experience varied.

In conclusion, DP is reasonable for treating pancreas-abutting RPS located at the LUQ when both complications and complete resection are considered.

## Data Availability Statement

The raw data supporting the conclusions of this article will be made available by the authors, without undue reservation.

## Ethics Statement

The studies involving human participants were reviewed and approved by Samsung Medical Center. Written informed consent for participation was not required for this study in accordance with the national legislation and the institutional requirements.

## Author Contributions

KK acquired, analyzed, and interpreted data and wrote the manuscript. KL designed the project, analyzed and interpreted data, and wrote the manuscript. JL analyzed and interpreted data and wrote the manuscript. SJ and SL acquired data. JH and JK analyzed and interpreted data. JP interpreted data. All authors contributed to the article and approved the submitted version.

## Conflict of Interest

The authors declare that the research was conducted in the absence of any commercial or financial relationships that could be construed as a potential conflict of interest.

## Publisher’s Note

All claims expressed in this article are solely those of the authors and do not necessarily represent those of their affiliated organizations, or those of the publisher, the editors and the reviewers. Any product that may be evaluated in this article, or claim that may be made by its manufacturer, is not guaranteed or endorsed by the publisher.

## Supplementary Material

Supplementary Figure 1 Comparison of OS and LRFS rates between the DP and non-DP group. (A) Comparison of OS between the DP and non-DP group. (B) Comparison of LRFS between the DP and non-DP group. Group comparisons were performed using Kaplan–Meier and log-rank tests. OS, overall survival; LRFS, local recurrence-free survival; DP, distal pancreatectomy.

## Abbreviations

BMI, body mass index; CT, computed tomography; DDLPS, dedifferentiated liposarcoma; DP, distal pancreatectomy; FNCLCC*, Fédération Nationale des Centres de Lutte Contre le Cancer;* ICU, intensive care unit; LMS, leiomyosarcoma; LR, local recurrence; LRFS, local recurrence-free survival; LUQ, left upper quadrant; MPNST, malignant peripheral nerve sheath tumor; OS, overall survival; PD, pancreaticoduodenectomy; POPF, postoperative pancreatic fistulas; RPS, retroperitoneal sarcoma; SFT, solitary fibrous tumor; TARPSWG, Trans-Atlantic Australasian Retroperitoneal Sarcoma Working Group; WDLPS, well-differentiated liposarcoma.
