# Supplementary material for: Postoperative Outcomes of Distal Pancreatectomy for Retroperitoneal Sarcoma Abutting the Pancreas in the Left Upper Quadrant
Source: Front Oncol. 2021 Dec 20;11:792943. doi: 10.3389/fonc.2021.792943 (PMC8721218; doi:10.3389/fonc.2021.792943)
Supplement: Supplementary file 1 [file DataSheet_1.docx]

**SUPPLEMENTARY TABLE 1 |** Univariate and multivariate analyses of risk factors associated

with survival.

| **Variables** | | **Univariate** | | **Multivariate** | |
| --- | --- | --- | --- | --- | --- |
|  |  | **HR (95% CI)** | **p-value** | **HR (95% CI)** | **p-value** |
| Age | | 1.02 (1.00–1.05) | 0.069 | 1.02 (0.99–1.05) | 0.151 |
| Male sex | | 1.19 (0.64–2.22) | 0.587 |  |  |
| BMI | | 1.01 (0.90–1.13) | 0.895 |  |  |
| Primary RPS | | 0.71 (0.37–1.36) | 0.298 |  |  |
| Tumor size | | 1.00 (1.00–1.01) | 0.911 |  |  |
| Resection of pancreas | | 1.54 (0.83–2.88) | 0.172 | 1.51 (0.77–2.98) | 0.232 |
| R2 resection | | 4.99 (2.50–9.97) | < 0.001 | 6.25 (2.98–13.14) | < 0.001 |
| DDLPS | | 1.14 (0.61–2.13) | 0.678 |  |  |
| FNCLCC histologic grade | |  |  |  |  |
|  | Grade 1 or 2 | 1 (Ref.) |  |  |  |
|  | Grade 3 | 3.46 (1.84–6.50) | < 0.001 | 3.95 (1.99–7.83) | < 0.001 |
| Severe complication | | 1.65 (0.82–3.32) | 0.161 |  |  |

*BMI,* body mass index; *RPS,* retroperitoneal sarcoma; *DDLPS,* dedifferentiated liposarcoma; *FNCLCC,* *Fédération Nationale des Centres de Lutte Contre le Cancer;* *HR,* hazard ratio, *CI* confidence interval.

**SUPPLEMENTARY TABLE 2 |** Univariate and multivariate analyses of risk factors associated

with local recurrence.

| **Variables** | | **Univariate** | | **Multivariate** | |
| --- | --- | --- | --- | --- | --- |
|  |  | **HR (95% CI)** | **p-value** | **HR (95% CI)** | **p-value** |
| Age | | 1.09 (0.59–2.03) | 0.783 |  |  |
| Male sex | | 1.73 (0.93–3.22) | 0.084 |  |  |
| BMI | | 0.96 (0.85–1.08) | 0.504 |  |  |
| rimary RPS | | 0.52 (0.28–0.98) | 0.043 | 0.51 (0.26–0.99) | 0.048 |
| Tumor size | | 1.00 (1.00–1.01) | 0.810 |  |  |
| Resection of pancreas | | 1.09 (0.59–2.03) | 0.783 | 0.94 (0.48–1.84) | 0.863 |
| R2 resection | | 2.68 (1.22–5.89) | 0.014 | 2.64 (1.19–5.88) | 0.017 |
| DDLPS | | 1.43 (0.76–2.67) | 0.267 |  |  |
| FNCLCC histologic grade | |  |  |  |  |
|  | Grade 1 or 2 | 1 (Ref.) |  |  |  |
|  | Grade 3 | 1.92 (1.01–3.65) | 0.047 | 1.97 (1.02–3.80) | 0.044 |

*BMI,* body mass index; *RPS,* retroperitoneal sarcoma; *DDLPS,* dedifferentiated liposarcoma; *FNCLCC,* *Fédération Nationale des Centres de Lutte Contre le Cancer;* *HR,* hazard ratio, *CI* confidence interval.

**SUPPLEMENTARY TABLE 3 |** Univariate and multivariate analyses of risk factors associated

with survival in patients who underwent DP.

| **Variables** | | **Univariate** | | **Multivariate** | |
| --- | --- | --- | --- | --- | --- |
|  |  | **HR (95% CI)** | **p-value** | **HR (95% CI)** | **p-value** |
| Age | | 1.02 (0.98–1.06) | 0.235 | 1.03 (0.99–1.08) | 0.162 |
| Male sex | | 0.91 (0.37–2.24) | 0.837 |  |  |
| BMI | | 1.04 (0.86–1.25) | 0.677 |  |  |
| Primary RPS | | 1.03 (0.40–2.64) | 0.997 |  |  |
| Tumor size | | 1.00 (1.00–1.01) | 0.540 |  |  |
| Microscopic pancreatic invasion | | 1.65 (0.65–4.19) | 0.288 | 2.18 (0.82–5.82) | 0.119 |
| R2 resection | | 11.4 (2.95–44.1) | < 0.001 | 6.48 (1.60–26.27) | 0.009 |
| DDLPS | | 1.46 (0.55–3.86) | 0.443 |  |  |
| FNCLCC histologic grade | |  |  |  |  |
|  | Grade 1 or 2 | 1 (Ref.) |  |  |  |
|  | Grade 3 | 4.73 (1.74–12.83) | 0.002 | 5.25 (1.74–15.88) | 0.003 |
| Severe complication | | 1.34 (0.51–3.53) | 0.560 |  |  |

*DP,* distal pancreatectomy; *BMI,* body mass index; *RPS,* retroperitoneal sarcoma; *DDLPS,* dedifferentiated liposarcoma; *FNCLCC,* *Fédération Nationale des Centres de Lutte Contre le Cancer;* *HR,* hazard ratio, *CI* confidence interval.
