# Supplementary figures and images for: Postoperative Outcomes of Distal Pancreatectomy for Retroperitoneal Sarcoma Abutting the Pancreas in the Left Upper Quadrant
Source: Front Oncol. 2021 Dec 20;11:792943. doi: 10.3389/fonc.2021.792943 (PMC8721218; doi:10.3389/fonc.2021.792943)

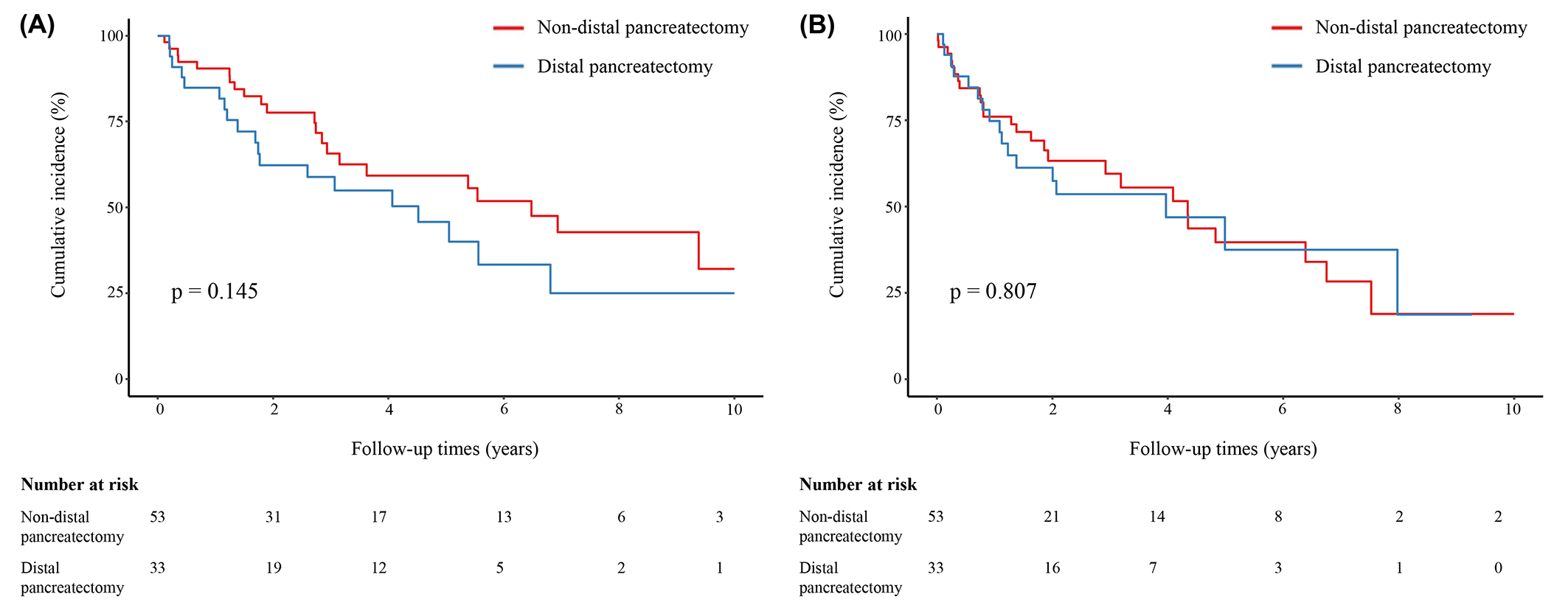

Supplement: Supplementary Figure 1 — Comparison of OS and LRFS rates between the DP and non-DP group. (A) Comparison of OS between the DP and non-DP group. (B) Comparison of LRFS between the DP and non-DP group. Group comparisons were performed using Kaplan–Meier and log-rank tests. OS, overall survival; LRFS, local recurrence-free survival; DP, distal pancreatectomy. [file Image_1.tif]
